# Ten simple rules for successfully completing a graduate degree in Latin America

**DOI:** 10.1371/journal.pcbi.1005682

**Published:** 2017-09-21

**Authors:** Ernesto Ruelas Inzunza, Gabriela I. Salazar-Rivera, Magdiel Láinez, María Guadalupe Ruiz-Gómez, Carlo A. Domínguez-Eusebio, Griselda Cristóbal-Sánchez, Issaac A. Teodosio Faustino, Edel Pérez-López, Meagan L. Campbell, Marcus Vinicius Merfa, Ivonne Tatiana Latorre Beltrán, Fernanda Armas, Claudio Mota-Vargas

**Affiliations:** 1 Instituto de Biotecnología y Ecología Aplicada, Universidad Veracruzana, Xalapa, Veracruz, Mexico; 2 Universidad Veracruzana, Centro de Investigaciones Tropicales, Xalapa, Veracruz, Mexico; 3 Facultad de Biología, Universidad Veracruzana, Xalapa, Veracruz, Mexico; 4 Department of Entomology and Plant Pathology, Auburn University, Auburn, Alabama, United States; 5 Independent researcher, Bogotá, Colombia; 6 Instituto de Ecología A.C., Xalapa, Veracruz, Mexico; Dassault Systemes BIOVIA, UNITED STATES

## Introduction

The successful completion of a graduate program in biological sciences is an endeavor that entails a passion for science. It requires some essential ingredients, such as the ability to think independently, a deep understanding of your study system, focus on strategic priorities, and establishing collaborations. A graduate program is also filled with challenges and hurdles. These and other key items have been covered in the literature, including papers in the “Ten Simple Rules” series [1, 2].

We have found, however, that in addition to those commonalities, students face regional variations, features that are specific to a particular part of the world. In this article, we posit that certain circumstances faced by graduate students are distinctively Latin American. On one side, limitations in human, infrastructure, and monetary resources are features that underlie the performance of the majority of graduate programs around the developing world [3]. On the other side, many unexplored ideas and research avenues dazzle the future possibilities of students of this region. Regardless, there is something unique to Latin American programs that needs to be explicitly said. We hope that the following 10 rules can appropriately summarize those characteristics.

The aim of this paper is to help prepare students interested in graduate programs in Latin America for the exciting experience they are about to start. When reading each of these rules, keep in mind the goal of this paper is to power your interest in science by presenting you its brilliant side as well as its caveats. We put special emphasis on how creativity, collaborations, and a great dose of energy can help them overcome those limitations.

## Rule 1: Investigate what you are getting into

Choose your adviser and your program carefully [1]. Graduate programs tend to have a local flavor and specialization. Consequently, many of them lack a critical mass in some areas. It is difficult to find a world expert in your specific field that—in addition to having similar research interests as yours—can provide funding or materials to support at least part of your work. Students often include a codirector for their graduate work, who usually brings additional expertise and other resources to their research but that often puts them in the midst of opposing views of how their own work should be done. However, 2 experienced views and 2 sources of financial support (even if meager) are better than 1.

If at all possible, meet your future adviser(s) in person. This is of vital importance. Culturally, these meetings are more valued than other forms of communication. If you don’t find an adviser with closely matching interests, a codirector is often the way to make 2 research topics meet.

Students entering a master’s degree normally initiate their program with a broader set of interests than students entering a doctoral program (for whom a closer focus is expected). In both cases, the refinements of their investigations will come with time and in the process of crafting a research project. Be open-minded and concede on some of your initial views of work, but do not get into doing something you don’t want to do. Enter a field you feel passionate about.

Figure out the Latin American cultural idiosyncrasies regarding students; learn about the habits of your adviser(s), how much time and attention they give to them, how willing they are to go out of their way to help their students, and how good they are at communicating. Generally speaking, senior, better-funded scientists have less time for students.

References of the specific program you are applying to are key. With very few standardized, region-wide, graduate program rankings (e.g., Brazil’s Fundação CAPES), a way you can gain insights into the program you are interested in is by researching its faculty, department, and institution using Google Scholar, ResearchGate, and Academia.edu. Word of mouth matters, especially in Latin America. Ask current or past students of the program about their experiences. Beware of (often private university–offered) “duckie” degrees. The basic infrastructure of an institution is a criterion of utmost importance, particularly if your research is demanding in terms of equipment, space, computing, and laboratory conditions. Consider having international committee members (often a great resource [4]).

For those interested in professional futures outside academia, there are very few programs preparing students for careers in private, non-profit, and government positions. The majority of the opportunities for growth in those sectors are accessed through your own enthusiasm, life experience, and hard work.

### Rule 2: Financial resources for graduate students are awarded on a competitive basis

Some countries have robust scholarship programs for graduate students, often open to applicants from any country (provided they pass through the regular admissions procedures). Funding is usually limited or highly competitive. Take advantage of these programs, as they are constantly adjusting the number of scholarships available and the amount of financial support that they provide. Some graduate programs (very few, we should say) have their own additional funding to supplement graduate students. Most scholarship programs are mutually exclusive—accepting one precludes you from applying to a second one. Working on other jobs while you are a graduate student, when allowed, is discouraged and often limited to a certain number of hours per week. In some countries, scholarships are actually student loans (the so-called “credit scholarships”).

Consider that the standard monthly scholarship is only sufficient to cover the expenses of a single person and that having dependents only makes you eligible for additional funds under some circumstances. Many students report using some of their scholarship funds to cover parts of their research expenses.

Learn how to get funds from external sources (Box 1). Go beyond the national boundaries for this; small grants are available from international, and even from nonconventional (e.g., private), sources for graduate students. Being a student member of scientific societies makes you eligible for larger pool of grants.

**Box 1. How to find funding for graduate student research in Latin America**

Looking for funding to cover your graduate research can take you to an online labyrinth. Below, we list 4 common gateways to find what you are looking for.

**Graduate fellowships.** The funding to cover the bulk of your graduate studies typically comes from fellowships offered by federal research agencies. Examples of in-country fellowship programs for graduate students are Mexico’s Consejo Nacional de Ciencia y Tecnología (http://conacyt.gob.mx/index.php/becas-y-posgrados), which offers fellowships for graduate studies and postdoctoral research in Mexico. These fellowships are available to applicants from any country or for Mexican nationals who plan to study abroad. In Argentina, the Consejo Nacional de Investigaciones Científicas y Técnicas (http://convocatorias.conicet.gov.ar/becas/) works similarly, with additional grants in support for research projects from graduate students. In Brazil, the Conselho Nacional de Desenvolvimento Científico e Tecnológico (http://www.cnpq.br/web/guest/apresentacao13/) supports graduate work. Many of these agencies have special opportunities for women in science, special projects, internationalization, and study abroad opportunities that are separate from their standard graduate fellowships.

**Scientific societies.** The majority of the most prominent scientific societies in the world have lists of grants and prizes available for students. Are you an ecologist? Check the website of the Ecological Society of America (https://www.esa.org/esa/careers-and-certification/funding-and-grants/). Perhaps an evolutionary biologist? Find the grants section of the Society for the Study of Evolution (http://www.evolutionsociety.org/). For earth and space scientists, the American Geophysical Union has funding listings as well (http://education.agu.org/grants/). Biochemists and molecular biologists can find funding opportunities in the American Society for Biochemistry and Molecular Biology website (https://www.asbmb.org/careers/scholarshipsandawards/). This list is a tiny sample. Check the society that most closely matches your field and take advantage of this benefit to its members.

**Courses, workshops, and conferences.** The organizers of training and academic exchange gatherings, such as conferences, symposia, and its associated courses and workshops, normally have funding available to facilitate graduate student attendance. This support has many different forms: registration fee waivers, free society memberships, volunteering opportunities, and competitive travel grants for attendants. Explore your possibilities carefully and put time into applying for relevant opportunities.

**Grants and prizes from subject-specific sources.** Last, many groups ranging from scientific to amateur make funding available for graduate students through small grant programs. Google them using the search words that best fit your research. Here is the top hit of the search string “grants + bird + research”: http://ornithologyexchange.org/resources/grants.html. It is a list of 246 sources of funding ranging from a few hundred dollars to several hundred thousand (with a median of about $4,000). Latin American students are eligible to many of them.

Some students entering graduate programs that already have academic positions can take a salaried leave of absence. If you are going back to grad school from a job elsewhere, remember that becoming a student is a very demanding commitment and, consequently, comes with changes in your income. Save money. Project future expenses. Be ready for periods where your savings will be your best bet. Graduate school is a full-time endeavor.

## Rule 3: Master administrative procedures from admission to graduation

Contrary to the somewhat standardized admissions processes at universities in the developed world, the process to select graduate students varies greatly in Latin American programs (often within the same university). Deadlines, requirements, exams, interviews, etc. are rarely the same across programs of the same discipline. This means that each and every application requires a substantial effort to put together.

Follow admission rules strictly. Be aware that some graduate programs include prep courses for their own degrees. Standard exams are often the most valued criterion for selecting students and a filter to reduce the size of the group of applicants.

Throughout the duration of your program, be meticulous when facing administrative procedures. Latin American graduate programs are plagued by paperwork requirements. The systems themselves can be rigid and not prone to creative solutions. When the specific requirements of a program do not match the paperwork that exists in your country, the first answer is frequently “no.” Be persistent and try to overcome that first no. Make in-person visits to program officers, get to know the registrar and administrators, be strict and exhaustive when compiling certificates, apostilles, notarized documents, immigration paperwork, and other forms, letters, and signatures. Your management of those procedures and requirements needs to be spotless. Plan way ahead of time and always meet deadlines.

Officers running graduate programs often do many jobs at once. We know programs run by people who have a research program of their own, teach, and coordinate the graduate program at a university or research center. Be persistent when communicating with them. Emails very often do not suffice. Insist. Get on the phone. Get answers to your questions. And do so with grace and good manners.

## Rule 4: The English language is essential

Let us say it again: It is essential [4, 5]. The overwhelming majority of your reading materials while you are a graduate student are in English. The language to communicate your results in peer-reviewed journals and reach broad audiences is English as well. The standard language used in international conferences and symposia is English. Many courses, software, listservs, newsletters, scientific societies, and a myriad of other tools to catapult your science career are, as you can imagine, in English. You will need to work hard in overcoming the limitation of English not being your native language. You won’t go very far without it. Better embrace it, learn English, and do it sooner rather than later.

With the English language as one of your skills, you should be able to access and navigate the somewhat flat field of science. The cost of not knowing English is too expensive, and you cannot afford it.

## Rule 5: Learn how to write

Papers are the most important communication currency in the sciences. Understand their elements: the main narrative, tables, figures, appendices, and other ancillary/supplementary information. Journals have adopted a variety of formats for the publication of research reports, opinion essays, letters, and other types of papers.

Many graduate programs have courses and training opportunities to learn how to write papers. These courses teach how to build a manuscript around a central idea, organize its content, write clear and concise messages, and be impeccable with regards to grammar, spelling, and punctuation. They also teach what, where, when, and how to publish.

Learning good writing is a complex process. It is another essential skill you must have to succeed as a scientist.

## Rule 6: Economic and logistical limitations are key criteria for selecting study subjects

Field site safety, social unrest, logistical complexity, distance to work sites, equipment availability, and qualified technical expertise are examples of central aspects that need to be carefully gauged when embarking on a research project. Many of them can be overcome with enough energy and time spent writing grants and establishing collaborations, but know that some items may just be way beyond what is reasonable or doable.

## Rule 7: Time is money or “El tiempo es oro”

Stay focused and solve—as soon as you can and in the best way possible—all those personal commitments that could distract from your work in the upcoming years. Figure out all housing, transportation, and family issues that can be solved ahead of time. Seek help or put away from yourself the additional headache of having to deal with problems that could have been solved ahead of time while being a graduate student. Learn about how to manage time, financial resources, and people as part of your training. Always bear in mind the expected standard duration of a doctoral program is 4 years and just 2 for a master’s program (Rule 1 in [2]).

It is true that family closeness and tightly knit social groups are much stronger in Latin America than in other regions of the world (and can be an amazing help), but they can also be a time, effort, and emotional distraction from your course of study. Do your best to find a balance of their costs and benefits. Many graduate students can overcome the heavy weight of being the pillar of the family, but only with Herculean efforts that almost invariably affect the quality and quantity of their graduate work.

## Rule 8: Make the best out of the courses you have at hand

Graduate programs in Latin America can be much broader in scope than you wish. Many of the programs, by necessity, have “fixed” courses that every student has to take and electives that aren’t quite as diverse and specialized as you need. Many programs have limitations on the number of courses you can take at other institutions (and issues over the compatibility of the evaluation point system arise quickly). Graduate programs often recognize such limitations and encourage you to exploit the collaboration connections of your university (some of them international [4]). Put extra time and effort into finding high-quality courses, even if they take you to faraway places. Find out if your university has ongoing formal agreements with other programs and make sure coursework offered elsewhere is compatible with your program. Find out if you need or can revalidate coursework taken at other places. Complete your coursework as soon as possible (you will need that time and energy in order to develop a strong research project).

## Rule 9: Give your own skills and potentials the right dimension

Be sure you are fair in dimensioning your own strengths and potentials. Become a true expert in your field! Know your stuff: Speak and write about it firmly, but don’t be cocky. Know that graduate students endure similar problems anywhere in the world; e.g., did you ever read about imposter syndrome [3] or writer’s block [5]? Low self-esteem, a common problem faced by students while in graduate school, seems to be particularly acute in Latin America.

**Fig 1:**
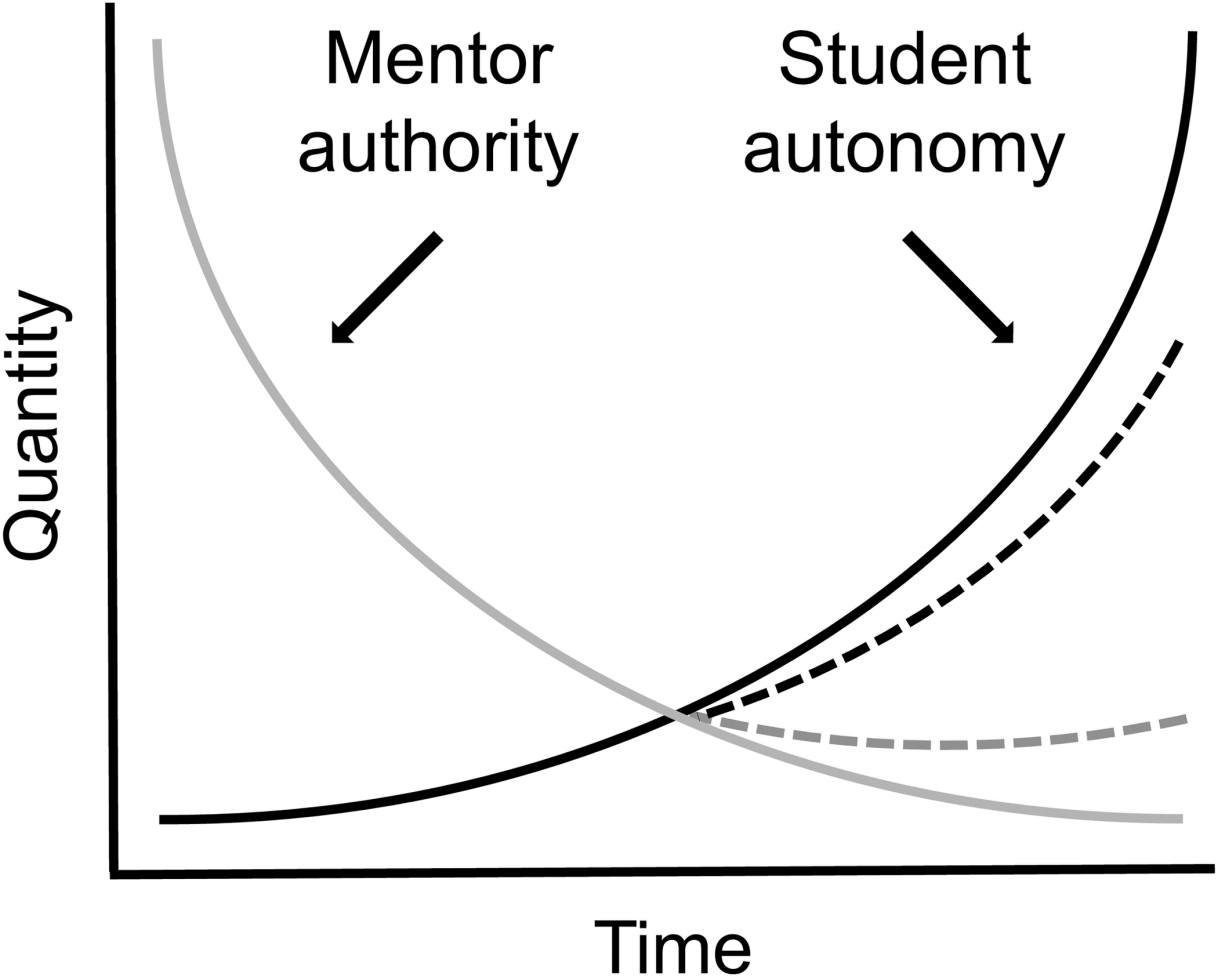
An equilibrium model of academic authority and autonomy.

Do not wait for your adviser to be the source of all initiative. Take the right position in the “autonomy/authority” continuum (Fig 1, Box 2). Follow Rule 2 in [1] closely. Being a graduate student is not a temporary job for your résumé; it is the preparation period for your career in science.

**Box 2. Academic autonomy and authority in graduate programs**

In graduate education, the academic authority of teachers and advisers decreases—or should decrease—as a function of time (Fig 1). In the figure above, this reduction in the role of mentors (illustrated as a gray curve) goes down as the autonomy of students (black curve) increases. The degree to which advisers concede such a gain in autonomy varies depending on the mentoring style, the strength of student skills, and the culture of the professor–student relationship. Conversely, students are able to attain autonomy of their graduate research depending on how strongly they feel about their own skills. These differences are illustrated in the model above by dotted lines that represent the variance of autonomy-authority curves. Graduate students are expected to gain a lot (in their master’s degrees) to almost absolute (in their doctoral degrees) autonomy throughout the course of their studies. One of the aspirations of advisers is that their advisees have gained substantial expertise in their field by the time of degree completion.

## Rule 10: Be proactive, creative, collaborative, and self-taught

You have in your hands the ability to clear obstacles you may find during your graduate program. Collaborate with other students and research scientists. Take advantage of the unprecedented opportunities to communicate, travel, take online courses, and learn from others in active exchanges via email both nationally and internationally [6]. Take a year abroad (it is a strong addition to your CV). Be open to criticism from your peers and professors. Ask about what you don’t know (the worst question is the one that is never asked). Circulate your proposals, papers, and ideas for comments and reciprocate the help that you have received from others.

Join scientific societies. Be active in their committees, attend their meetings, and volunteer at their conferences and symposia; present your research data there, get feedback, and network with other students and professionals. These experiences are crucial in your professional development.

In most Latin American graduate programs, obtaining teaching skills is not part of your graduate training. If you are seeking a career in academia, make sure you find a way to get this experience.

Learn the basic tools of the trade in your discipline: equipment, analysis techniques, software, etc. Engage, on your own or in groups, in self-study and in journal/book clubs.

## A corollary

Latin American graduate programs have made great strides in producing excellent graduates and future scientists. Now you can find internationally outstanding and highly productive research groups in many countries. Many foreign students come to Latin American graduate programs to enjoy a rich cultural experience.

Perhaps the greatest reward of completing a graduate degree in Latin America is that many (most?) of the study systems haven’t been as thoroughly studied as in the developed world. Your research can make giant discoveries and provide the very first insights into a particular question or be the benchmark work that finds the solution to a big problem. You can make a difference by becoming a finely trained mentor that guides the careers of many more young scientists and professionals, influencing policy, governance, and public opinion.

Make a commitment to pay back to the country and people that helped you to do what you’ve done as part of your graduate research. Besides publishing all of your research, those investment returns can take the form of actions to bridge your work with issues of public interest: papers for a general readership, manuals, catalogues, and conferences for a broader audience. Some of us think the social meaning of Latin American science should privilege applied (over basic) research and focus on the solution of society’s most poignant problems (others do not, but that’s okay: basic science, when properly conducted and published, will prove useful to mundane applications at some point in the future). Avoid academic endogamy. Consider changing to an entirely different program, institution, city, and/or country when you are ready to your next step (a PhD if you are a MSc student, or a postdoctoral position). Make the broadening of your horizons a priority of your training.
